# Absurd and Dangerous Prescriptions

**Published:** 1892-02

**Authors:** 


### ABSURD AND DANGEROUS PRESCRIPTIONS.

It is very much the *fashion* now to publish prescriptions. It is too much the fashion with many doctors to adopt those he sees published. There are still many medical men who believe disease to be an entity, to be driven out of the system, and that certain medicines are "good for" certain diseases.

Knowing that the formulæ printed in leading medical journals, especially those bearing the name of DaCosta or Pepper or Roosa or some other well known practitioner, will be adopted by many doctors, editors should be careful in admitting them to their pages, and should be equally careful in reading proof to see that they are correctly printed. Fatal results may follow the giving of medicine on the authority of a formula copied from a medical journal. We often, in looking over the published prescriptions, find excessive doses of some strong drug, or a dose advised, totally disproportioned to the age of the patient, if a child.

These remarks are suggested in seeing the following prescription in the *St. Louis Clinique* for Jan:

"Croup: Dr. Johnson uses the following:

℞ Aquae dist .................................... ℥i
Potas Chlorate,
Potas Iodid ................................ aa ʒi
Emuls arabicæ ................................ ℥ii
Mucilag acaciæ,
Extr ipecac fl,
Olei copaibæ ................................ aa ℥i

"M. Sig: Shake well. Dose, a teaspoonful every ten minutes to an infant of eight months till free vomiting ensues, and then continue the same dose every half hour or hour till the disease is cured" [or, we will add, till the child is vomited to death.—ED].

We do not know who "Dr. Johnson" is, but any man who would give an infant of eight months ten drops of the fluid extract of ipecac and ten drops of oil of copaibæ every ten minutes should be prosecuted. Supposing (an impossibility) the infant should retain it, in one hour he will have taken six doses or 60 minims of each (a teaspoonful); and as the dose is to be continued every half hour after "free vomiting" has set in, the effect would be to keep the child vomiting indefinitely, and it would never have a chance to get "cured."

If, as one would suppose, the mixture is intended as a prompt emetic, and the object free vomiting, what on earth does "Dr. Johnson" want with the iodide and chlorate of potassium? They can possibly do no good, as they are to be vomited immediately; and if an emetic be the object, there are a thousand substances known to act promptly and to be unattended with danger, which should be preferably used. Then why publish such an absurd mixture, even if the doses were even approximately correct? Again, will some one please inform us what "Emuls Arabicæ" may be? And if, as one would suppose, it is made of "gum arabic" why order a mucilage of the same?

That Dr. Porter, the intelligent editor of the *Clinique*, should have published such an absurd and dangerous prescription can only be ascribed to inattention or inadvertence.

If correctly quoted, "Dr. Johnson" has written himself "an ass."

